# Facial asymmetry outcome of orthognathic surgery in mild craniofacial microsomia compared to non-syndromic class II asymmetry

**DOI:** 10.1007/s00784-024-05899-6

**Published:** 2024-08-28

**Authors:** Yun-Fang Chen, Frank Baan, Stefaan Bergé, Yu-Fang Liao, Thomas Maal, Tong Xi

**Affiliations:** 1 Department of Craniofacial Orthodontics, Chang Gung Memorial Hospital, Taipei, Taiwan; 2 Craniofacial Center, Chang Gung Memorial Hospital, Taoyuan, Taiwan; 3 Craniofacial Research Center, Chang Gung Memorial Hospital, Linkou, Taiwan; 4 Radboudumc 3D Lab, Radboud University Medical Center, Nijmegen, The Netherlands; 5 Department of Oral and Maxillofacial Surgery, Radboud University Medical Center, Geert Grooteplein 10, Nijmegen, 6500 HB The Netherlands; 6 Department of Craniofacial Orthodontics, Chang Gung Memorial Hospital, Taoyuan, Taiwan; 7 College of Medicine, Chang Gung University, Taoyuan, Taiwan

**Keywords:** Craniofacial microsomia, Class II asymmetry, Facial asymmetry, Orthognathic surgery

## Abstract

**Objectives:**

To compare the facial asymmetry after bimaxillary surgery between mild craniofacial microsomia (CFM) and non-syndromic class II asymmetry.

**Materials and methods:**

Cone-beam computed tomography scans of adults with Pruzansky-Kaban types I and IIA CFM (CFM groups, *n* = 20), non-syndromic skeletal class II asymmetry (Class II group, *n* = 20), and normal controls (control group, *n* = 20) were compared. The area asymmetry of lower face and jaw bones was quantified. Landmark-based method was used to evaluate the lower facial asymmetry regarding midline, cants, and contour.

**Results:**

There were no significant postoperative differences in the hemi-facial and hemi-jaw area asymmetry between CFM and Class II groups, both of which were significantly larger than the control group. No significant difference was found in the midline deviation and lip and occlusal cants between CFM and Class II groups. The vertical contour asymmetry in CFM group became significantly larger than Class II group. Compared to the control group, the deviation of pronasale, subnasale, and soft-tissue menton, lip and occlusal cants, and sagittal and vertical contour asymmetry in CFM group were significantly larger, as were the deviation of subnasale and soft-tissue menton and vertical contour asymmetry in Class II group.

**Conclusions:**

The vertical contour asymmetry of mild CFM was significantly larger than non-CFM class II after surgery, while the area asymmetry, midline deviation, cants, and sagittal contour asymmetry of lower face showed no significant difference.

**Clinical relevance:**

Be aware that correcting vertical asymmetry of contour, lip, and dentition in CFM is still challenging.

## Introduction

Craniofacial microsomia (CFM) is known for the hypoplastic and deformed maxillomandibular complex and ears [1, 2]. The Pruzansky-Kaban system (types I, IIA, IIB, and III) is the most widely used tool for classification of the principal defect of CFM, that is, the mandibular deformity. In contrast to severe CFM (types IIB and III), the small (types I and IIA) and misshapen (type IIA) mandibles of mild CFM (types I and IIA) yet retain sufficient temporomandibular joint position and function [3]. Orthognathic surgery (OGS) at skeletal maturity is an efficient option for mild CFM to address the two main chief complaints of facial asymmetry and skeletal class II malocclusion [4–6]. Early surgical intervention on jaws should be considered only when encountering breathing or psychosocial problems, as the inherent asymmetric pattern was found to persist or resume during the remaining growth period [7–12].

In the previous study assessing skeletal characteristics [2], compared to cases of non-CFM class II deformity presenting similar extent of chin deviation, mild CFM cases (types I and IIA) did have significantly larger size asymmetry in the mandibular body, ramus and condyle and more prominent roll asymmetry in the maxillomandibular complex. Also, asymmetric curvature of the lateral contour was specifically found in the mandibular body of the mild CFM group. Given that the asymmetry of the maxillomandibular structures in CFM were more severe than in non-CFM class II asymmetry, a less satisfactory OGS outcome might be expected for CFM cases. According to the results of Chen et al. [13, 14], the facial asymmetry in non-CFM class II was significantly improved after OGS; however, the final chin deviation (2.64 mm) was still obvious compared to normal controls. On the other hand, though the OGS outcome of CFM evaluated with three-dimensional (3D) data was reported to be satisfactory and the remaining chin deviation ranged from 0.5 to 2.5 mm, they are case series studies and thus of low evidence level [5, 6, 15].

The aim of this study was to compare the lower facial asymmetry outcome of bimaxillary OGS between mild CFM and non-syndromic class II asymmetry. The null hypothesis was that the facial asymmetry after surgery showed no significant differences between CFM and Class II groups. Furthermore, the facial asymmetry of the two groups after surgery was compared with normal controls to evaluate objectively the effectivity of the treatment.

## Materials and methods

This study was approved by the Institutional Ethics Committee of the hospital (202102196B0C101). Due to the retrospective nature of this study, the committee waived the requirement for written informed consent. This study protocol conformed to the Declaration of Helsinki.

### Study groups

Adults (> 16 years old) with unilateral CFM and skeletal class II asymmetry who visited Chang Gung Craniofacial Center between 2010 and 2018 for a combination of orthodontic and two-jaw orthognathic treatment were consecutively selected in this retrospective study based on the following criteria. CFM group: (1) Pruzansky-Kaban type I or IIA CFM with a deviated chin toward the affected side, (2) absence of craniofacial anomalies other than CFM, (3) no history of maxillomandibular surgery or trauma, (4) Le Fort I osteotomy and bilateral sagittal split osteotomy advancement surgery by the attending surgeons who were in the same craniofacial team and were trained by the same most experienced surgeon of the center, (5) completion of postsurgical orthodontic treatment, and (6) availability of cone-beam computed tomography (CBCT) scans before OGS (T1) and at orthodontic debonding (T2). Class II group consisted of subjects with non-syndromic skeletal class II (ANB angle > 4°) presenting significant skeletal menton deviation that matched the severity of that of CFM group and meeting the same 3rd to 6th inclusion criteria as CFM group.

### Control group

Control subjects were selected consecutively from adults who had pre-treatment CBCT images at the same center for other dental indications (e.g., implants, third molars, dental crowding, or spacing). Two orthodontists and one surgeon evaluated the profiles of the control subjects. When a unanimous decision was reached with regard to a harmonious profile, the subject was included in the control group. The exclusion criteria for the control group were: (1) craniofacial anomalies, (2) significant facial asymmetry (menton deviation > 2 mm), (3) anterior open bite, or severe dental crowding or spacing, or (4) history of craniofacial surgery or trauma. The control group was used to generate 3D norms.

### CBCT image acquisition and 3D cephalometry

CBCT images of the head of all subjects were obtained in natural head position using an i-CAT 3D Dental Imaging System (Imaging Sciences International, Hatfield, PA, USA) with parameter settings of 120 kV, 0.4 mm isotropic voxel size, 40 s scan time, and 16 cm × 16 cm field of view.

The CBCT scans at T1 were reconstructed toward virtual 3D head models and orientated in Maxilim^®^ software (Medicim NV, Mechelen, Belgium) based on reference landmarks including orbitale and porion on the non-deviated side, bilateral frontozygomatic points, nasion, and sella. Three reference planes were subsequently automatically generated: a horizontal reference plane passing through sella and 6 degrees below the sella-nasion plane, a midsagittal plane passing through sella and nasion and perpendicular to the horizontal reference plane, and a coronal plane passing through sella and perpendicular to the horizontal and midsagittal planes [16, 17]. The transverse, sagittal, and vertical directions correspond to the x-axis, y-axis, and z-axis, respectively. A positive coordinate value indicated the posterior, superior, and left sides.

The CBCT scan at T2 was registered on the corresponding CBCT at T1 using voxel-based method at the anterior cranial base. The deviated side was defined as the side of the chin deviation and where CFM was present. Before cephalometric analysis, the images of 3D heads were flipped if needed so that the deviated side was located on the left side for all subjects. The same was done to Class II group. Cephalometric landmarks were then identified on the virtual 3D head models with the aid of multiplanar views for measurements (Tables 1 and 2).

**Table 1:** 3D cephalometric landmarks

Landmark	Symbol	Definition
Pronasale	Prn	The most anterior midpoint of the nasal tip
Subnasale	Sn	The midpoint on the nasolabial soft-tissue contour between the columella crest and the upper lip
Labiale superius	Ls	The midpoint of the vermilion line of the upper lip
Soft-tissue menton	Me’	The most inferior midpoint on the chin located at the level of the 3D cephalometric hard-tissue menton
Cheilion^a^	Ch	The point located at each labial commissure
Upper canine^a^	U3	The tip of the maxillary canine
Upper first molar^a^	U6	The mesiobuccal cusp tip of the maxillary first molar

^a^ Bilateral landmarks

**Table 2:** Craniofacial asymmetry measurements

Measurements	Definition
**Linear measurement**,** mm**	
Prn deviation	The transverse distance between pronasale and the midsagittal plane
Sn deviation	The transverse distance between subnasale and the midsagittal plane
Ls deviation	The transverse distance between labiale superius and the midsagittal plane
Me’ deviation	The transverse distance between soft-tissue menton and the midsagittal plane
**Angular measurement**,** degrees**	
Ch cant	The angle between the line connecting bilateral cheilions and the horizontal reference plane projected on the coronal reference plane
U3 cant	The angle between the line connecting bilateral upper canines and the horizontal reference plane projected on the coronal reference plane
U6 cant	The angle between the line connecting bilateral upper first molars and the horizontal reference plane projected on the coronal reference plane
**Area measurement**,** %**	
Hemi-facial area asymmetry	The absolute value of 1 minus the ratio of bilateral areas (deviated side/non-deviated side) which was bordered by the soft-tissue outline, the midsagittal plane, and a horizontal plane passing through Mid-Nose point^a^
Hemi-jaw area asymmetry	The absolute value of 1 minus the ratio of bilateral areas (deviated side/non-deviated side) which was bordered by the skeletal outline, the midsagittal plane, and a horizontal plane passing through Mid-Nose point^a^

^a^ The midpoint of the line connecting soft-tissue nasion and subnasale

### Calculation of area asymmetry of lower face and jaw bones

**Fig. 1:**
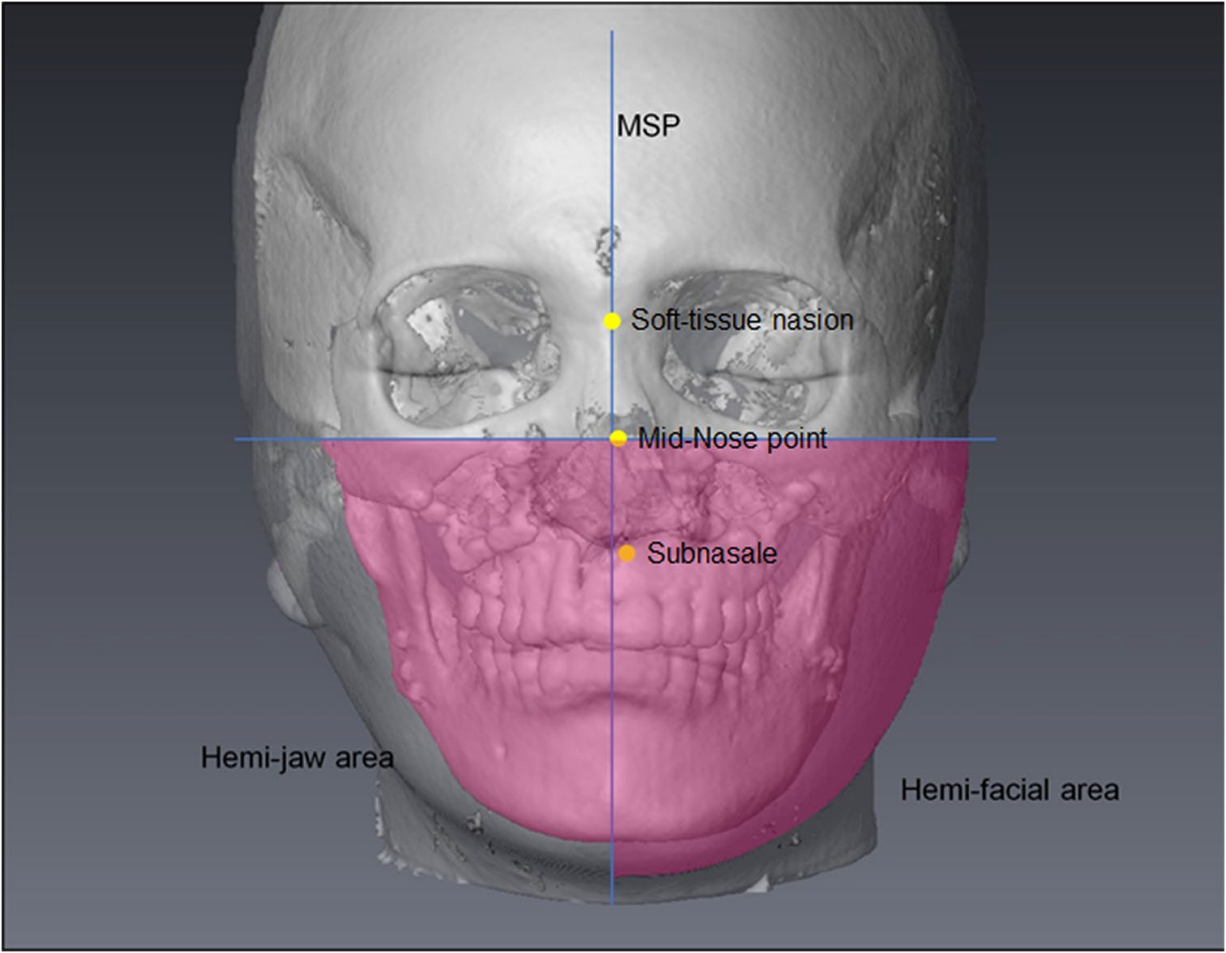
Measurements of 2D hemi-facial and hemi-jaw areas, which are the areas below a horizontal plane passing through Mid-Nose point. MSP, the midsagittal plane of the 3D head. Mid-Nose point, the midpoint of the line connecting soft-tissue nasion and subnasale

Images in the frontal view were captured from Maxilim^®^, and Image J software (version 1.53t for Macintosh, National Institutes of Health, Bethesda, Md) was used to trace the target areas manually to measure the hemi-facial and hemi-jaw areas of the lower face of each subject at T1 and T2 (Table 2; Fig. 1). The area asymmetry was calculated using the following formula:

$$\text { area asymmetry }=\left|1-\frac{\text { area of deviated side }}{\text { area of non deviated side }}\right|$$

A larger value means a more severe asymmetry between bilateral areas.

### Calculation of contour asymmetry of lower face

**Fig. 2:**
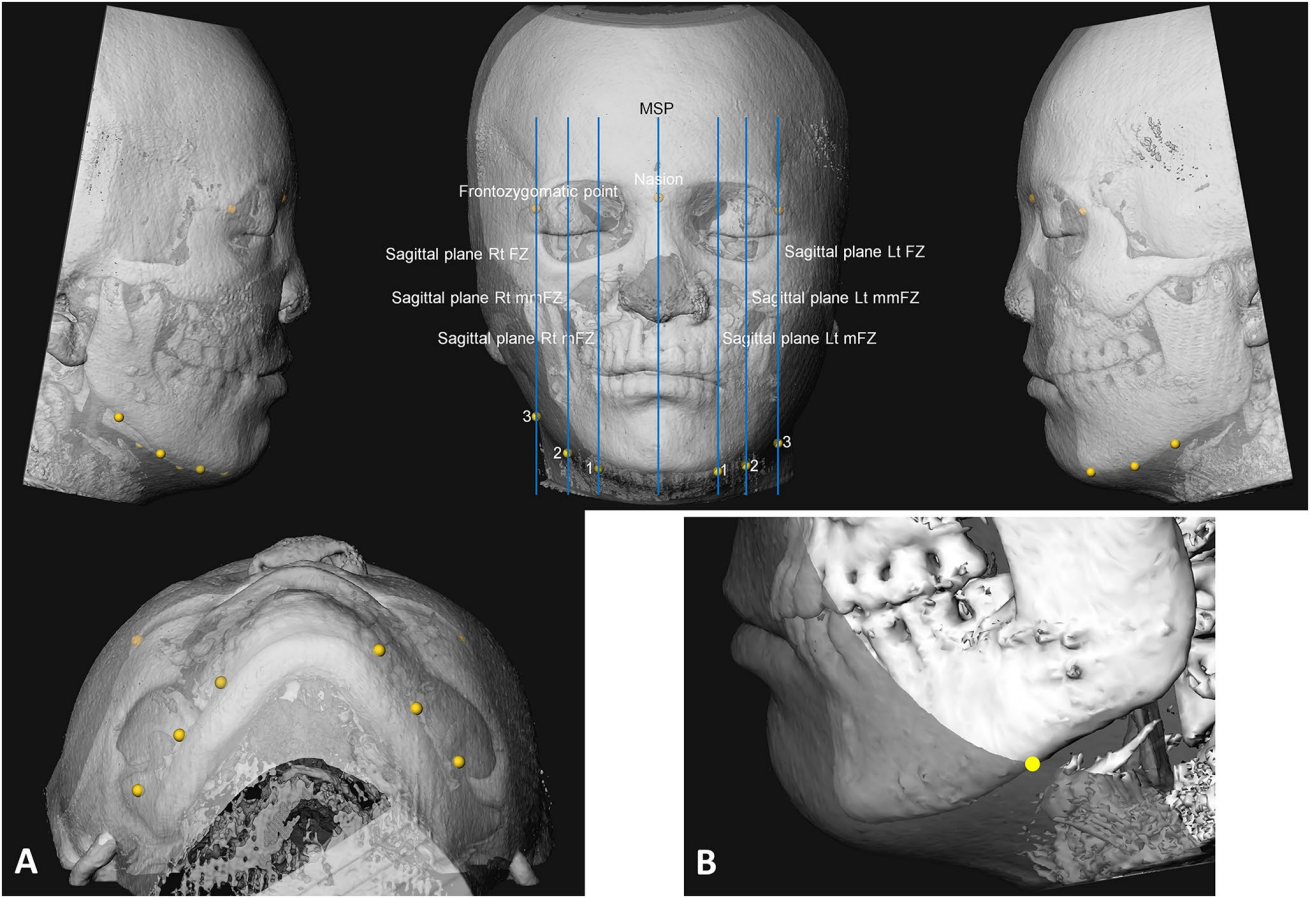
Soft-tissue contour analysis of lower face by using 3DMedX software. **A** the three reference points (nasion and frontozygomatic points) and seven sagittal planes used to define the three soft-tissue contour points on each side of face; **B** a soft-tissue contour point assigned on one reference sagittal plane from the lateral view as the intersection point between soft-tissue surface and mandibular lower border. For the definitions of the reference planes and soft-tissue contour points, please refer to Table 3

All the registered pairs (T1 and T2) of 3D head models were imported into 3DMedX software (v1.2.30.0, 3D Lab Radboudumc, the Netherlands) for contour assessment. A semi-automatic program developed by the 3D Lab was used. The skeletal reference landmarks (nasion and frontozygomatic points) were annotated on the 3D head of T1 and referred to the 3D head of T2. Subsequently, several sagittal planes were automatically created based on the reference landmarks. These planes were used for defining soft-tissue contour landmarks to quantify the asymmetry of bilateral soft-tissue contour (Table 3; Fig. 2).

**Table 3:** Skeletal landmarks and sagittal planes for defining soft-tissue contour landmarks, and the contour measurements

	Definition
Skeletal reference landmark (manually assigned)	
Nasion	The midpoint of the frontonasal suture
Frontozygomatic point^a^	The most anterior point of the frontozygomatic suture
Plane (automatically generated)	
Midsagittal plane	The midsagittal plane passing through sella and nasion and perpendicular to the horizontal reference plane described in the text
Sagittal plane Rt FZ	A sagittal plane passing through the frontozygomatic point on the right side
Sagittal plane Lt FZ	A sagittal plane passing through the frontozygomatic point on the left side
Sagittal plane Rt mFZ	A sagittal plane in the center between the midsagittal plane and Sagittal plane Rt FZ
Sagittal plane Lt mFZ	A sagittal plane in the center between the midsagittal plane and Sagittal plane Lt FZ
Sagittal plane Rt mmFZ	A sagittal plane in the center between Sagittal plane Rt FZ and Sagittal plane Rt mFZ
Sagittal plane Lt mmFZ	A sagittal plane in the center between Sagittal plane Lt FZ and Sagittal plane Lt mFZ
Contour point (manually assigned)	
Soft-tissue contour point 1^a^	Assigned on Sagittal plane mFZ from the lateral view, the intersection point between soft-tissue surface and mandibular lower border
Soft-tissue contour point 2^a^	Assigned on Sagittal plane mmFZ from the lateral view, the intersection point between soft-tissue surface and mandibular lower border
Soft-tissue contour point 3^a^	Assigned on Sagittal plane FZ from the lateral view, the intersection point between soft-tissue surface and mandibular lower border
Measurement	
Mean sagittal position of soft-tissue contour^a^	(y1 + y2 + y3)/3
Mean vertical position of soft-tissue contour^a^	(z1 + z2 + z3)/3
Sagittal asymmetry of soft-tissue contour	Absolute value of [(y1d – y1nd) + (y2d – y2nd) + (y3d – y3nd)]
Vertical asymmetry of soft-tissue contour	Absolute value of [(z1d – z1nd) + (z2d – z2nd) + (z3d – z3nd)]

^a^ Bilateral landmarks or measurements

^b^ On the deviated side, soft-tissue contour point 1 (x1d, y1d, z1d), soft-tissue contour point 2 (x2d, y2d, z2d), and soft-tissue contour point 3 (x3d, y3d, z3d). On the non-deviated side, soft-tissue contour point 1 (x1nd, y1nd, z1nd), soft-tissue contour point 2 (x2nd, y2nd, z2nd), and soft-tissue contour point 3 (x3nd, y3nd, z3nd)

The primary outcome variables were the facial asymmetry measurements. The primary predictor was the type of mandibular asymmetry deformity (mild CFM vs. non-CFM class II asymmetry). The secondary outcome variables were the correlations of the change (∆T2-T1) of soft-tissue menton (Me’) with the changes of the cants of lip and upper canines and first molars and the area asymmetry of lower face and jaw bones.

To assess the intra-investigator reliability, the CBCT measurements were conducted by one investigator for 10 randomly chosen patients twice, with an interval of two weeks. To assess the inter-investigator reliability, a second investigator independently conducted the same process for the same CBCT dataset.

### Statistical analysis

According to our previous studies (Chen 2019, Chen 2022) [2, 13], a minimum of 17 subjects per group should be recruited. This sample estimation was using G-Power software (version 3.1.9.7; Franz Faul, University of Kiel, Kiel, Germany) with an effect size of 1.0, a significance level of 5% (*p* < 0.05), and a power of 80%.

The Statistical Package for Social Sciences for Windows 24 (SPSS 24, IBM Corp., NY, USA) was used for statistical analyses. Differences between two of the three groups (CFM, Class II, and control groups) were compared using a Mann-Whitney U test or chi-square test where indicated. Differences between data of T1 and T2 of a group were assessed using a Wilcoxon sign-rank test. Spearman’s correlation coefficients were used to assess the correlation between two continuous variables; the correlation coefficients between 0.4 and 0.7 were considered as moderate correlation. Intra-class correlation (absolute, two-way mixed) was calculated to assess the intra- and inter-observer reliability. All statistical tests were two-sided, and the level of significance was set at 0.05.

## Results

### Subject characteristics

Based on the selectin criteria, 25 candidates were initially selected for each of CFM and Class II groups. Mann-Whitney U test showed that the menton deviation before treatment was significantly larger in CFM group than Class II group. To obtain a comparable menton deviation in both groups, subjects with the most severe menton deviation in CFM group were excluded and those with the least severity in the Class II group were also excluded. This process resulted in 20 subjects in each group with no significant difference in menton deviation between them, along with another 20 subjects enrolled in the control group. The subject characteristics are described in Table 4. Fifteen CFM subjects were of Pruzansky-Kaban type I and five were of type IIA. Before treatment, the median values of Me’ deviation of CFM and Class II groups were significantly larger than the control group (7.9 mm vs. 0.9 mm, *p* < 0.001; 7.6 mm vs. 0.9 mm, *p* < 0.001). The median time of evaluation after OGS was 17.9 (range, 10.1 to 49.4 months) and 18.2 months (range, 8.4 to 35.2 months) for CFM and Class II groups, respectively. For CFM group, the improvement of ANB didn’t reach statistical significance (5.3° ◊ 3.2°, *p* = 0.065), and the resultant ANB showed no significant difference from the control group. For Class II group, the ANB showed significant improvement (4.9° ◊ 2.7°, *p* = 0.003) and thus had no significant difference from the control group after treatment (Table 5).

**Table 4:** Subject characteristics of 3 groups

Median (IQR)	CFM group, *n* = 20	Class II group, *n* = 20	Control group, *n* = 20	*p*, CFM vs. Control	*p*, Class II vs. Control
Age at T1, years	18.7 (18.2, 20.2)	26.4 (24.3, 30.6)	25.8 (20.4, 31.0)	0.001	0.410
Age at T2, years	21.5 (19.8, 22.6)	28.6 (25.8, 32.5)	25.8 (20.4, 31.0)	0.026	0.068
Female, n	14	16	16	0.716	1.000
Pruzansky-Kaban type I, n	15	-	-	-	-
Pruzansky-Kaban type IIA, n	5	-	-	-	-
Post-OGS treatment time of orthodontics, months	17.9 (14.8, 25.9)	18.2 (14.5, 25.7)	-	-	-

*IQR*, interquartile range; *T1*, before treatment; *T2*, at orthodontic debonding

**Table 5:** Comparison of deformities of 3 groups before and after treatment

Median (IQR)	CFM group, *n* = 20			Class II group, *n* = 20			*p*, CFM_T1 vs. Class II_T1	*p*, CFM_T2 vs. Class II_T2	Control group, *n* = 20	*p*, CFM_T1 vs. Control	*p*, CFM_T2 vs. Control	*p*, Class II_T1 vs. Control	*p*, Class II_T2 vs. Control
	T1	T2	*p*, T1 vs. T2	T1	T2	*p*, T1 vs. T2							
ANB, degrees	5.3 (3.1, 6.0)	3.2 (2.4, 5.2)	0.065	4.9 (3.1, 7.0)	2.7 (1.6, 4.4)	0.003	0.654	0.380	4.0 (2.4, 4.6)	0.087	0.551	0.039	0.129
SNA, degrees	78.9 (75.1, 82.9)	81.0 (76.4, 82.9)	0.001	81.9 (80.2, 84.2)	81.1 (80.3, 82.6)	0.091	0.015	0.465	80.2 (78.0, 84.6)	0.151	0.635	0.311	0.597
SNB, degrees	75.8 (71.4, 79.9)	77.5 (72.8, 81.5)	0.033	77.3 (75.2, 79.0)	78.1 (77.1, 80.1)	0.009	0.441	0.337	76.6 (75.3, 80.3)	0.167	0.465	0.703	0.279
Hemi-facial area asymmetry, %	6.2 (4.0, 10.5)	5.4 (1.6, 10.9)	0.409	7.4 (3.3, 16.3)	4.2 (2.3, 7.3)	0.033	0.698	0.565	1.8 (0.9, 5.3)	0.001	0.026	0.001	0.028
Hemi-jaw area asymmetry, %	9.4 (4.2, 11.8)	7.6 (2.9, 16.1)	0.927	7.3 (3.3, 20.2)	6.0 (1.9, 11.5)	0.143	0.883	0.383	2.1 (0.5, 4.5)	0.001	0.005	0.002	0.035
Prn deviation, mm	0.9 (0.4, 2.0)	1.3 (0.5, 2.0)	0.957	1.2 (0.4, 1.7)	1.0 (0.3, 1.8)	0.730	1.000	0.379	0.5 (0.4, 0.8)	0.070	0.004	0.038	0.079
Sn deviation, mm	1.0 (0.6, 2.3)	1.0 (0.5, 2.3)	0.373	1.2 (0.7, 2.1)	0.9 (0.6, 1.3)	0.120	0.815	0.615	0.6 (0.3, 0.8)	0.008	0.013	0.002	0.021
Ls deviation, mm	1.5 (1.1, 2.6)	1.1 (0.3, 2.6)	0.084	1.9 (0.8, 3.5)	1.3 (0.3, 2.4)	0.026	0.753	0.973	0.6 (0.3, 1.1)	< 0.001	0.081	< 0.001	0.064
Me’ deviation, mm	7.9 (3.4, 13.2)	2.6 (1.4, 5.6)	0.005	7.6 (5.2, 11.7)	4.3 (1.8, 5.3)	< 0.001	0.551	0.654	0.9 (0.4, 1.2)	< 0.001	< 0.001	< 0.001	< 0.001
Ch cant, degrees	4.2 (2.4, 5.8)	2.1 (0.9, 4.2)	0.001	3.3 (1.7, 4.9)	1.5 (0.9, 2.3)	< 0.001	0.490	0.297	0.8 (0.5, 1.5)	< 0.001	0.025	< 0.001	0.097
U3 cant, degrees	6.1 (4.8, 7.7)	2.3 (1.4, 2.8)	< 0.001	4.0 (2.5, 6.5)	1.4 (0.4, 3.0)	< 0.001	0.045	0.198	1.0 (0.3, 2.2)	< 0.001	0.021	< 0.001	0.343
U6 cant, degrees	6.0 (4.0, 7.9)	1.9 (1.3, 2.7)	< 0.001	3.9 (2.7, 5.3)	1.5 (0.6, 3.1)	< 0.001	0.041	0.351	1.2 (0.6, 1.7)	< 0.001	0.007	< 0.001	0.189
Sagittal asymmetry of soft-tissue contour, mm	11.6 (2.1, 26.3)	17.3 (6.7, 27.5)	0.546	22.7 (14.0, 34.8)	13.4 (4.3, 20.7)	< 0.001	0.043	0.327	6.7 (4.2, 9.3)	0.149	0.009	< 0.001	0.086
Vertical asymmetry of soft-tissue contour, mm	11.6 (3.5, 23.3)	14.0 (9.0, 25.2)	0.216	8.0 (4.5, 15.7)	7.5 (5.0, 11.2)	0.475	0.327	0.021	1.5 (0.9, 3.8)	< 0.001	< 0.001	< 0.001	< 0.001

IQR, interquartile range; T1, before treatment; T2, at orthodontic debonding

### Measurement reliability

Intra-observer reliability, analyzed by the intraclass correlation coefficient (ICC), was excellent (mean ICC, 0.999; 95%CI, 0.981–1.000). Inter-observer reliability was excellent (mean ICC, 0.997; 95%CI, 0.967–1.000).

### Area asymmetry of lower face and jaw bones

Before and after treatment, there was no significant difference in the hemi-facial and hemi-jaw area asymmetry between CFM and Class II groups (Table 5).

Regarding hemi-facial area asymmetry, there was no significant change in the asymmetry (6.2% ◊ 5.4%, *p* = 0.409) after treatment for CFM group, and the preoperative (*p* = 0.001) and postoperative (*p* = 0.026) asymmetry both were significantly larger than that of the control group. In contrast, for Class II group, the asymmetry significantly decreased (7.4% ◊ 4.2%, *p* = 0.033), but however was still significantly larger than that of the control group (1.8%, *p* = 0.028).

Regarding hemi-jaw area asymmetry, for CFM and Class II groups, there was no significant change, and the asymmetry was still significantly larger than that of the control group (7.6% vs. 2.1%, *p* = 0.005; 6.0% vs. 2.1%, *p* = 0.035) after treatment.

In CFM group, there was a significant correlation between the hemi-facial and hemi-jaw area asymmetry before (ρ = 0.571, *p* = 0.008) and after treatment (ρ = 0.845, *p* < 0.001). A significant correlation was shown between preoperative and postoperative hemi-facial area asymmetry (ρ = 0.659, *p* = 0.002), and not shown in the skeletal part (ρ = 0.150, *p* = 0.527). In Class II group, there was a significant correlation between the hemi-facial and hemi-jaw area asymmetry before (ρ = 0.940, *p* < 0.001) and after treatment (ρ = 0.779, *p* < 0.001). No significant correlation was found between preoperative and postoperative asymmetry. For both CFM and Class II groups, there was no significant correlation between the changes (∆T2-T1) in the Me’ deviation and area asymmetry (Table 6).

**Table 6:** Spearman’s correlation of the change of soft-tissue menton deviation with the changes of variables of cants and area asymmetry

	∆Me’ deviation	∆Ch cant	∆U3 cant
**CFM group**			
∆Ch cant	ρ = 0.598, *p* = 0.005		
∆U3 cant	ρ = 0.753, *p* < 0.001	ρ = 0.720, *p* < 0.001	
∆U6 cant	ρ = 0.690, *p* = 0.001	ρ = 0.760, *p* < 0.001	ρ = 0.845, *p* < 0.001
∆Hemi-facial area asymmetry	ρ = 0.347, *p* = 0.145		
∆Hemi-jaw area asymmetry	ρ = 0.179, *p* = 0.464		
**Class II group**			
∆Me’ deviation			
∆Ch cant	ρ = 0.863, *p* < 0.001		
∆U3 cant	ρ = 0.814, *p* < 0.001	ρ = 0.919, *p* < 0.001	
∆U6 cant	ρ = 0.758, *p* < 0.001	ρ = 0.789, *p* < 0.001	ρ = 0.798, *p* < 0.001
∆Hemi-facial area asymmetry	ρ = 0.114, *p* = 0.631		
∆Hemi-jaw area asymmetry	ρ = 0.143, *p* = 0.548		

∆, T2-T1; *Me’*, soft-tissue menton; *Ch*, cheilion; *U3*, tip of upper canine; *U6*, mesiobuccal cusp tip of upper first molar

### Soft-tissue midline deviation, and lip and occlusal cants

Before treatment, the median values of pronasale (Prn), subnasale (Sn), labial superius (Ls), Me’ deviation and cheilion (Ch) cant between CFM and Class II groups showed no significant difference, and all were significantly larger than those of the control group (all *p* < 0.05) except for the Prn deviation of CFM group. Meanwhile, the median values of upper canine (U3) cant and upper first molar (U6) cant in CFM group were significantly larger than Class II group, which were all significantly larger than those of the control group (Table 5).

After treatment, no significant difference was found in the extents of all soft-tissue midline deviation, and lip and occlusal cants between CFM and Class II groups. For CFM and Class II groups, there were significant improvements in the deviation of Me’ and cants of Ch, U3 and U6; the deviation of Ls significantly improved in Class II group. In CFM group, the deviation of Prn, Sn, Me’ and the cants of Ch, U3, U6 was significantly larger than those of the control group. In Class II group, the deviation of Sn and Me’ was significantly larger than those of the control group.

In both CFM and Class II groups, there were significant positive correlations between all the changes (∆T2-T1) in the Ch cant, U3 cant, U6 cant, and Me’ deviation (all *p* < 0.01). The correlation was moderate to high in CFM group and high in Class II group (Table 6).

### Contour asymmetry of lower face

**Fig. 3:**
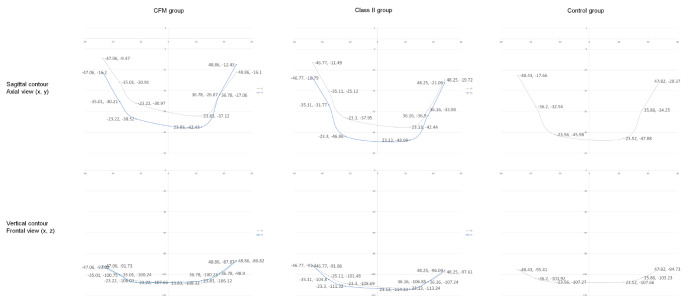
2D line plots of the median values of the soft-tissue contour points (three points on each side) before (gray line) and after (blue line) treatment for CFM, Class II, and the control groups to visualize the contour asymmetry

Before treatment, the median value of sagittal asymmetry of soft-tissue contour was significantly smaller in CFM group than Class II group (11.6 mm vs. 22.7 mm, *p* = 0.043). After treatment, the median value of vertical asymmetry of soft-tissue contour was significantly larger in CFM group than Class II group (14.0 mm vs. 7.5 mm, *p* = 0.021) (Table 5; Fig. 3).

After treatment, in CFM group, the sagittal and vertical asymmetry in the soft-tissue contour insignificantly worsened and were significantly larger than that of the control group (sagittal, 17.3 mm vs. 6.7 mm, *p* = 0.009; vertical, 14.0 mm vs. 1.5 mm, *p* < 0.001).

After treatment, in Class II group, the sagittal contour asymmetry significantly improved (22.7 mm ◊ 13.4 mm, *p* < 0.001) and became no significant different from the control group; the vertical asymmetry was still significantly larger than that of the control group (7.5 mm vs. 1.5 mm, *p* < 0.001).

After treatment, in CFM group, the contour on the non-deviated side became significantly more anteriorly positioned (-21.0 mm ◊ -29.0 mm, *p* < 0.001). In Class II group, the contour on the deviated side became significantly more anteriorly (-32.4 mm ◊ -34.6 mm, *p* = 0.006) and superiorly (-106.8 mm ◊ -105.1 mm, *p* = 0.030) positioned, and the contour on the non-deviated side became significantly more anteriorly (-23.7 mm ◊ -33.4 mm, *p* < 0.001) (Table 7).

**Table 7:** The mean position^a^ of soft-tissue contour

Median (IQR)	CFM group, *n* = 20			Class II group, *n* = 20		
	T1	T2	*p*, T1 vs. T2	T1	T2	*p*, T1 vs. T2
Mean sagittal position of soft-tissue contour, deviated side, mm	-26.4 (-34.1, -20.3)	-27.8 (-33.4, -20.3)	0.090	-32.4 (-35.8, -27.7)	-34.6 (-40.2, -31.4)	0.006
Mean sagittal position of soft-tissue contour, non-deviated side, mm	-21.0 (-30.5, -13.6)	-29.0 (-38.9, -24.7)	< 0.001	-23.7 (-29.3, -15.1)	-33.4 (-38.5, -25.4)	< 0.001
Mean vertical position of soft-tissue contour, deviated side, mm	-98.8 (-102.1, -93.3)	-97.5 (-100.0, -93.1)	0.261	-106.8 (-111.3, -101.9)	-105.1 (-108.8, -98.8)	0.030
Mean vertical position of soft-tissue contour, non-deviated side, mm	-100.1 (-109.2, -94.3)	-98.6 (-109.1, -96.6)	0.261	-99.2 (-105.8, -95.4)	-102.6 (-108.4, -95.6)	0.053

IQR, interquartile range; T1, before treatment; T2, at orthodontic debonding

^a^ Please refer to Table 3 for the definition

## Discussion

The purpose of this study was to compare the asymmetry outcome of lower face after bimaxillary OGS between CFM and non-CFM class II asymmetry. The bilateral facial areas, midline landmarks, cant landmarks of lips and teeth, and contour landmarks, which are the most easily perceivable features of facial asymmetry by patients, were evaluated for analysis. To the authors’ knowledge, this is the first study performing systemic analysis of OGS outcome for mild CFM and further comparison with non-CFM class II asymmetry. The results of this study revealed the vertical asymmetry of soft-tissue contour remained significantly larger in CFM group compared to Class II group after treatment. Therefore, the null hypothesis was rejected. On the other hand, the initially more severe occlusal cants at the maxillary canines and first molars in CFM group got statistically significant improvement via OGS and showed no statistically significant difference compared to the treatment outcome of Class II group.

Perfect facial symmetry barely exists. Mild facial asymmetry is commonly observed in general population. In the current study, a group of subjects with harmonious faces was recruited to provide realistic standards for judging treatment outcome of patients with significant facial asymmetry. The extents of midline deviation and cant asymmetry (Table 5) were accordant with those reported in the literature (< 2 mm and < 4 degrees, respectively) [18–20]. Though the ANB angle of this control group seems to be slightly beyond the commonly-known mean value of 2 degrees for skeletal Class I relationship, it remained within the norm range (3.25° ± 1.43° for male and 3.42° ± 1.69° for female) proposed for Taiwanese population [21].

After treatment, all of the variables evaluating facial asymmetry showed no significant differences between CFM and Class II groups, except for the vertical asymmetry in the soft-tissue contour. For CFM group, the vertical difference between bilateral soft-tissue contour became larger after treatment, though the change didn’t reach statistical significance. And for clinicians, this worsening is an expected finding in CFM cases. Accordantly, it was mentioned in the study of Yamaguchi et al. [5] that for cases with prominent soft- tissue hypoplasia, OGS can exacerbate the soft-tissue discrepancy along the jawline on the deviated side due to the shifting and depletion of the underlying skeletal support and the stretching out of the soft tissue. As shown in our previous study [2], the height of mandibular body on the deviated side was significantly hypoplastic than that on the non-deviated side, implying that the contour generally would not be symmetric after centering the mandible, and therefore additional hard- or soft-tissue maneuver should be planned and consulted with patients beforehand. On the other hand, although the final vertical contour asymmetry of Class II group was significantly smaller than CFM group, OGS seemed to be ineffective in improving the soft-tissue contour asymmetry even for non-CFM class II subjects which was still distinguishable from normal controls. This was in agreement with the results of our previous study [14]. The morphology of the mandibular body for cases of non-CFM class II asymmetry showed only minimal asymmetry [2], and consequently another notable contributing factor for the final vertical asymmetry of the soft-tissue contour could be the residual positional asymmetry of the osteotomy segment. This reasoning is supported by the study of Chen et al. [14]. The soft-tissue thickness might also play a role in the contour asymmetry and should be studied in the future.

For each face, three pairs of contour landmarks were analyzed. To assess the severity of facial contour asymmetry, the differences of sagittal and vertical positions of each paired contour landmarks were calculated (Table 5). On the other hand, the mean value of the coordinates of three contour landmarks on each side of face was calculated to indicate the direction of contour movement after OGS. Thus, these mean positional values did not necessarily reflect the extent of contour asymmetry (Table 7). Plot images were used to illustrate the trends in asymmetry severity and directional movements of the soft-tissue contour (Fig. 3). Notably, for Class II group, the soft-tissue contour was bulkier on the deviated side than on the non-deviated side (Table 7; Fig. 3). This asymmetry improved significantly after OGS and, subsequently, the sagittal contour asymmetry showed no significant difference from the control group postoperatively (Table 5). In contrast, the sagittal contour asymmetry in CFM group, initially comparable to the control group (Table 5), slightly worsened after OGS, unfortunately becoming significantly more asymmetric than the control group. This could be attributed to the significant anterior movement of the contour on the non-deviated side (Table 7; Fig. 3). The depressed facial appearance on the deviated side after OGS in CFM is a problem that frequently confronts clinicians. While it might not be evident in frontal photographs, it usually can be perceived by professionals or even lay people in person. Fat grafting on the deviated side had been adopted in our center and worldwide [22] for CFM cases to increase the soft tissue projection and symmetry immediately after OGS or as a secondary procedure 6 months later, and satisfactory outcome was reported [5].

In CFM group, Prn deviation became prominent after treatment when compared to the control group. The deterioration was insignificant, and 1.25 mm of deviation is clinically acceptable (< 2 mm). Hajeer et al. [23] also reported nasal tip asymmetry development after OGS. One possible explanation is that to correct the prominent U3 and U6 cants in CFM while holding the upper dental midline in the center, intentional tilting the upper part of the maxilla would be necessary and thus impact the nasal tip symmetry. For both CFM and Class II groups, there was no significant change in Sn deviation; Ls deviation became more symmetric and thus showed no significant difference from the control group; and Me’ deviation got significant improvement via OGS but was still pronounced. Despite the final Me’ deviation showed no significant difference between the two groups, to our surprise the outcome was better in CFM. This might be explained by that faced with the questionable outcome of bilateral symmetry in CFM, a more accurate repositioning of the chin midline would be highly demanded.

Roll asymmetry is a feature of CFM [2]. Fortunately, the cants of Ch, U3, and U6 of CFM group all got significant improvement via OGS and were below 2.5 degrees, although still significantly severer than the control group. Cants less than 4 degrees can be considered as clinical acceptable [18, 20]. For Class II group, the final extents of cants approached the normal range and showed no significant difference from the control group. The correlation between the improvement in lip cant and chin midline deviation has been a consistent finding for class I [24] and class III asymmetry [25]. And the current study showed that this correlation also applied to class II asymmetry and further even to CFM patients.

For both CFM and Class II groups, although no statistical correlation was found between the extents of Me’ deviation and area asymmetry, either before or after treatment (data are not shown), and between the changes of Me’ deviation and area asymmetry as well. When the Me’ deviation in CFM and Class II groups improved after OGS, there was a corresponding enhancement in area symmetry in both the lower face and jaw bones. This parallel improvement in Me’ deviation and facial asymmetry is logical and can be expected in advance in patients with symmetrically shaped mandibles. The current study further demonstrated that even for aberrantly shaped mandibles of mild CFM cases, centering the position of the mandible is still an important principle of OGS that clinicians can follow. It is worth noting that the hemi-facial and hemi-jaw area asymmetry in CFM could improve after OGS centering the midline structures, though the improvement was not significant. This could be the basis for secondary interventions to achieve a more ideal facial soft-tissue outcome. Considering the hemi-facial hypoplastic nature of CFM, restoring bilateral facial or jaw symmetry is hardly achievable solely via OGS. A combination of tissue augmentation on the deviation side and reductive intervention on the non-deviated side is usually recommended, because pure augmentation might excessively strain the hypoplastic soft-tissue envelope. In Class II group, the improvement in hemi-jaw area asymmetry was insignificant while the hemi-facial area asymmetry showed significant improvement, indicating a favorable soft-tissue response in response to hard-tissue correction. In CFM group, there was a high correlation (ρ = 0.845, *p* < 0.001) between the hemi-facial and hemi-jaw area asymmetry after treatment. This suggested that improving the underlying jaw symmetry could benefit the overlying soft-tissue facial symmetry, which has long been the treatment principle adopted by many clinicians. A moderate positive correlation (ρ = 0.659, *p* = 0.002) between preoperative and postoperative hemi-facial area asymmetry in CFM group implied that cases with more severe preoperative facial area asymmetry might have a lower chance to obtain a symmetric outcome. However, such a predictive correlation was not observed for non-CFM class II asymmetry cases.

There were limitations to this study. First, the number of subjects in each group was insufficient to draw a robust conclusion. Second, besides the innovative evaluation method of contour, we focused only on shifts and cants. Assessment of yaw asymmetry was conducted in the previous study [2], and ramus asymmetry in CFM could be a topic in future studies. Additionally, the median value of age of CFM group was significantly lower than that of Class II group, because most CFM subjects had regular appointments at our craniofacial center since childhood. Thus, OGS was planned when subjects reached skeletal maturity as indicated by stable body height and 2D cephalometry. Although residual mandibular growth could be a confounding factor, correlation tests showed no significant influence of age on the postoperative changes in the menton position in both groups (Spearman’s correlation tests, *p* = 0.400 to 0.596). This suggests that the role of age or residual growth is limited in the current study.

## Conclusions

After bimaxillary OGS, patients with mild CFM exhibited a significantly greater vertical contour asymmetry compared to patients with non-CFM class II asymmetry. No significant differences were observed in the area asymmetry of lower face and jaw bones, midline deviation of nose, lip, and chin, cants of lips and teeth, and sagittal contour asymmetry of the lower face between the two groups. Nevertheless, notably, the lip and occlusal cants of CFM cases were still significantly more prominent than normal controls. There were significant positive correlations between the changes in chin deviation and changes in lip and occlusal cants. No correlation was found between changes in chin deviation and area asymmetry.

## Data Availability

No datasets were generated or analysed during the current study.
